# Predicting Longitudinal Decline in Everyday Functioning Using Cognitive Challenge Measures

**DOI:** 10.1002/alz70857_101165

**Published:** 2025-12-24

**Authors:** Diane D. Zheng

**Affiliations:** ^1^ University of Miami School of Medicine, Miami, FL, USA

## Abstract

**Background:**

The predictive utility of proactive semantic interference (PSI) and failure to recover from proactive semantic interference (frPSI) deficits on the longitudinal everyday functional decline on the Clinical Dementia Rating Sum of Boxes (CDR‐SOB) among older adults with amnestic mild cognitive impairment (aMCI) has not been evaluated.

**Method:**

This longitudinal prospective study included 97 older adults, aged 54 to 98 years, who were diagnosed with amnestic mild cognitive impairment (aMCI) following a baseline evaluation at the 1Florida Alzheimer's Disease Research Center. The average age of participants was 71.7 years (SD 7.7), with 56% identifying as Hispanic and 51% as male. The mean education level was 15.7 years (range 4 – 22), and the mean MMSE score at baseline was 28.0. Participants were followed annually for 3 to 4 visits, with an average follow‐up period of 38.9 months (range 22.7–70.3 months). Comprehensive clinical and neuropsychological assessments, including the CDR‐SOB, were conducted during the annual visits. Amyloid PET scans were performed for 88 participants (91%), of whom 36 (41%) were amyloid‐positive (A+) and 52 (59%) were amyloid‐negative (A−). Latent growth curve modeling was used to estimate the trajectory of the CDR‐SOB. The associations between PSI, frPSI, and the CDR‐SOB growth curve trajectory were examined through a series of hierarchical conditional growth curve models.

**Result:**

The growth curve model that best fits the CDR‐SOB trajectory was a linear form and included the fixed and random effect of intercept and slope of time. After adjusting for age, sex, education, Hispanic background, Hopkins Verbal Learning Test (HVLT) immediate and delayed recall, and amyloid positivity, frPSI (β = ‐0.134, se=0.04, *p* <0.01) remained statistically significant in predicting a steeper slope on the trajectory of decline in CDR‐SOB.

**Conclusion:**

Baseline frPSI predicted the rate of everyday functional decline over time among participants with aMCI, independent of amyloid status. These findings highlight the utility of frPSI in predicting longitudinal change in CDR‐SOB.

